# The Use of Tablet-Based Multiple-Pass 24-Hour Dietary Recall Application (MP24Diet) to Collect Dietary Intake of Children under Two Years Old in the Prospective Cohort Study in Indonesia

**DOI:** 10.3390/nu11122889

**Published:** 2019-11-27

**Authors:** Min Kyaw Htet, Umi Fahmida, Tran Thanh Do, Michael J. Dibley, Elaine Ferguson

**Affiliations:** 1 South East Asian Ministers of Education Organization, Regional Center for Food and Nutrition (SEAMEO RECFON), Pusat Kajian Gizi Regional Universitas Indonesia, Salemba Raya 6, Jakarta 10430, Indonesia; umifahmida@gmail.com; 2 Sydney School of Public Health, Sydney Medical School, The University of Sydney, Sydney NSW 2006, Australia; michael.dibley@sydney.edu.au; 3 Sinergi Qalbu Fikri, Depok 16952, Indonesia; 4 National Institute of Nutrition, Hanoi 116110, Vietnam; thanhdo.tran@gmail.com; 5 London School of Hygiene and Tropical Medicine, London WC1E 7HT, UK; Elaine.Ferguson@lshtm.ac.uk

**Keywords:** dietary assessment, Indonesia, tablet-based dietary application, young children

## Abstract

Dietary intake data are crucial for developing or evaluating nutrition interventions to improve the nutritional status of populations. The collection of accurate and reliable dietary data in developing countries, however, remains challenging. The emergence of new technologies, which facilitate electronic data capture, might address some of these challenges. This paper aims to describe an application developed to collect a multiple-pass 24-h dietary recall, using electronic data capture, and compare the results to those estimated using a paper-based method. In this study, a tablet-based application was developed, in the CommCare platform, to evaluate the effectiveness, for improving dietary adequacy, of a package of behavior change interventions to reduce stunting and anemia among 6–23-month-old children in East Java, Indonesia (Baduta project). Dietary intakes of energy and nutrients were estimated using electronic data capture in the cohort study of the Baduta project (*n* = 680). We compared these results with those estimated using paper-based data capture in the project’s end-line cross-sectional study (*n* = 2740). We found a higher percentage of children classified as acceptable energy reporters (reported energy intake within the 95% CI of Total Energy Expenditure) with the electronic data capture compared with paper-based data capture (i.e., 60.8%, 72.4% and 80.7% for 6–8-, 9–11- and 12–23-month-old children, respectively, vs. 40.9%, 56.9%, and 54.3%, respectively). The estimated mean energy and nutrient intakes were not significantly different between these dietary data capture methods. These results suggest dietary data collection, using a tablet-based application, is feasible and can improve the quality of dietary data collected in developing countries.

## 1. Introduction

Rapid changes in dietary patterns in low- and middle-income countries (LMICs) are contributing to an increased risk of micronutrient deficiencies, stunting, overweight, and obesity [1]. To monitor these changes over time and develop effective interventions, the public health sector urgently needs accurate and reliable dietary data. One constraint has been the time and skill required to collect high-quality dietary data. In this regard, the development of a dietary assessment tool that standardizes interviews and reduces data processing time would play an important role in generating the evidence needed to address malnutrition in LMICs.

The use of electronic data collection, via mobile phones or tablets, has recently gained attention. Several studies have shown its advantages over paper-based data collection [2,3]. Other studies have shown it is also feasible, in LMICs, to collect dietary data via electronic data capture [4,5]. These applications, however, have not used an iterative process of data entry, which the recommended multiple-pass 24-h diet recall method requires [6]. Unlike structured questionnaires or single–pass data entry [4,5,7], direct entry of a multiple-pass 24-h dietary recall is a complex process that requires special data capture features [6]. There is computer-based software (GloboDiet) with the required features for collecting multiple-pass 24-h dietary recall data in Asia [8]. However, there would be advantages in having a tablet or mobile phone application for collecting multiple-pass 24-h recall data.

Another major challenge, when collecting retrospective dietary data, is the accurate estimation of food portion sizes. A systematic bias in the estimation of food portion sizes can result in under- or over-reporting of energy and nutrient intakes. Well-designed electronic data capture software can help reduce this error by displaying the respondent’s total energy intake at the end of the multiple-pass 24-h recall interview. This function, however, requires pre-loaded food composition data for all foods consumed. Given the diversity of dietary culture and patterns in South East Asia, the preloading of all food items before data collection is challenging. Therefore, an application needs to accommodate new foods that are not in its internal food composition table.

Progress has been made to improve the reliability and accuracy of dietary assessment methods in high-income countries; however, there is only limited research on this topic in LMICs. Given the advantages of electronic dietary data capture and the need for a reliable, field-friendly tool, for use in LMICs, we developed a tablet-based application (MP24Diet) for collecting multiple-pass repeated 24-h recall data in a longitudinal cohort study in Indonesia. In this paper, we describe the application and compare dietary intakes of energy and nutrients estimated using this application with those estimated using the conventional paper-based approach.

## 2. Methods

### 2.1. Study Context

In this study, we developed a tablet-based electronic data capture application to collect dietary data in a sub-study of the Baduta project [9]. The Baduta study was a community-based cluster randomized controlled trial conducted in the Sidoarjo and Malang districts of West Java to assess the impact of a package of behavior change interventions to improve infant and young child feeding practices, growth, and anemia. Baduta in the Indonesian language means children under two years old, and the aim of this study was to improve nutritional status, especially stunting and anemia among children under two years in Indonesia. This study consisted of two assessments: (1) baseline and end-line cross-sectional surveys of 0–23-month-old children conducted in February 2015 and 2017, respectively (*n* = 2435 at baseline and 2740 at end-line), and (2) a cohort study in which we recruited pregnant women from March–June 2015 and followed their infants until they were 24 months old (*n* = 680). In both the cross-sectional and cohort studies, dietary data were collected for 6–23-month-old children, using the multiple-pass 24-h recall. However, data were collected using a conventional paper-based approach in the cross-sectional surveys, and with electronic data capture in the cohort study. In this paper, we report the findings from dietary data collected from the cohort study and the end-line, cross-sectional study.

### 2.2. Development of the Electronic Data Collection Tool for 24-h Dietary Recall

We developed the electronic data collection application using the CommCare platform version v2.20 application (www.commcarehq.org). CommCare is an open-source code software and an extension to the JavaROSA codebase (code.javarosa.org) that can operate on a wide range of Java-enabled phones and tablets [10]. This platform was selected because it supports the iterative process required for the multiple-pass 24-h dietary recall. Specifically, in the multiple-pass 24-h recall, an uninterrupted recall of the diet for the whole day is collected (pass 1) before asking for details of the individual foods and beverages listed (pass 2) and their portion sizes and recipes (pass 3), followed by a summary of all foods and beverages recalled (pass 4). The application does not require many modules since the “repeat group” function in CommCare allows us to include as many food items as the respondent consumed and the “lookup” function allows us to search for a given food item in the food composition table (FCT) integrated into the app. We used Samsung Galaxy Tab S2 9.7 inch for data collection. See the website for further information (https://web.facebook.com/MP24Diet).

### 2.3. Steps in Data Collection Using the Application

**Figure 1:**
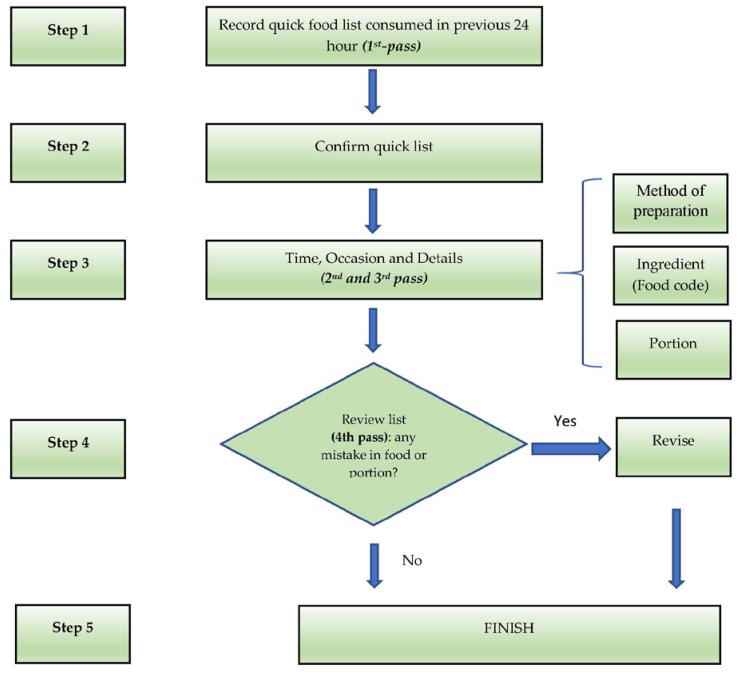
The flow of multi-pass 24-h dietary data collection using the application. Note: the quick list will be displayed throughout the interview to remind the remaining food items.

On opening the application, the user selects a participant from the case list (names of respondents) displayed on the tablet. Once the case is selected, the participant is asked to recall all foods and beverages consumed over the previous day (Figure 1; pass 1). After entering this quick food list, the enumerator asks details about the type of meal and time of consumption which they enter directly into the application. The user categorizes the meals as a single ingredient/food or a homogenous recipe. In the next pass, details of each food are sequentially recorded, including the time of consumption, local name, method of preparation, and other food-specific details (Figure 1; pass 2). The user selects a food code by searching for the food using one of three options: (1) searching by food group or subgroup, (2) entering the food code directly (if the food code is known), or (3) searching by name (fuzzy function). This step is critical to linking the food to its corresponding food composition data in the food composition table (FCT). Only the energy content of food items is embedded in the application in order to save memory and increase data processing speed. In the next pass, the participant is asked to estimate the portion size of each food/beverage recorded (Figure 1; pass 3). Afterward, the enumerator summarizes all the foods and recipes reported to confirm they correctly represent the child’s food intake for the previous day (Figure 1; pass 4). The energy contributed by each food item to the total dietary energy content (kcal) is also displayed, as well as the total energy intake from all food items. This function provides a quality control check at the end of the interview, i.e., for potential under- or over-reporting of energy intakes.

### 2.4. Portion Size Estimation

The enumerators were trained to follow a strict protocol for portion size estimation, which included (1) direct weighing of a similar food from respondents in real-time, (2) direct weighing of the equivalent volume of staple foods brought during the interview, and (3) by using the food album integrated into the app. For staple foods, enumerators carried samples of staple foods such as rice, noodle, and bread along with them, and the respondents were requested to estimate the equivalent amount of staple foods they had consumed. The indicated amount of staple food was then weighed by the enumerator to obtain the estimated portion size. This approach helped respondents to give the closest estimate of staple foods that they had consumed.

They weighed (Tanita digital scale for kitchen use, model KD-160, precision ±1 g; Tanita Corporation, www.tanita.com) the estimated amount of each food item consumed using food samples when an appropriate sample was available, or a food photo album, which was integrated into the application when food samples were not available. The food samples included staple foods carried by the enumerators (i.e., rice, noodles, and bread), foods available in the household, or foods purchased during the interview. We based the food photo album on the 2014 Indonesian “Total Diet Study” food photo album with the addition of foods specific to the study area. We used one picture per food item for most foods, but for some food items, there were 3 to 5 pictures for the different portion sizes available. Each photo showed the gram weight equivalent for the food portion shown, as well as a common household utensil (e.g., spoon, plate) to show scale.

### 2.5. Calculation of Energy and Nutrient Intakes

Energy and nutrient intakes were calculated using the Indonesian FCT data (www.panganku.org). We completed this FCT, in which there were missing nutrient values, with nutrient values from FCT data of other countries, including Singapore, Malaysia, Thailand, and the USA, after adjusting for differences in water content [11].

For standard recipes, the energy and nutrient contents were in the CommCare application’s FCT. For non-standard recipes, we estimated the nutrient content from the breakdown of its ingredients and the amount consumed in grams of each ingredient. If a recalled food item or recipe ingredient was not in the application’s internal FCT, the enumerators entered a standard code, i.e., 999, to identify the new food item. They immediately reported this new food item to their field supervisor, who reported it to the investigators. The investigators added the food into the application, after which the enumerators updated the application in their tablets to include the new foods. In this way, we updated all newly identified food items in the application’s internal FCT on an ongoing basis.

### 2.6. Paper-Based 24-h Dietary Recall

Conventional paper-based multiple-pass 24-h dietary recalls were used, in the cross-sectional survey, to collect dietary intake data for 6–23-month-old children. The questions, series of passes, and methods used to estimate food portions were identical to those used in the electronic data capture application. All enumerators, in both sub-studies, were trained in the same way.

### 2.7. Realistic Energy Ranges for the Children

To identify potential under- or over-reporting of energy intakes at the end of the electronic data capture interview, enumerators compared the estimated intakes of energy with an acceptable energy range for a child of the same age and breastfeeding status (Table 1). We derived these energy intake ranges from data describing the energy requirements (±2 SD) of children, which, for breastfed children, were also adjusted for energy intakes from breast milk (i.e., 413, 379, 346 kcal/day for 6–8, 9–11 and 12–23 months, respectively) as reported in Dewey and Brown (2003) [12].

### 2.8. Training of Enumerators

We recruited diploma III nutrition graduates from the local Polytechnic University as nutrition data enumerators for this study. They were trained to use the tablet-based electronic data capture system to collect 24-h recall data. During the training, the enumerators tried out the system before data collection commenced. Each interview took approximately 30–40 min. 

### 2.9. Ethical Approval

The Ethics Committee in the Faculty of Public Health, Universitas Indonesia, and the Human Research Ethics Committee at the University of Sydney provided ethical approval for the BADUTA study. Before data collection, we obtained written, informed consent from each respondent. The infant’s primary guardian (i.e., the infant’s mother) gave written informed consent for the infant’s participation in this study.

### 2.10. Data Analysis

The proportion of respondents who under- or over-reported their child’s energy intake was calculated using the predicted Total Energy Expenditure (pTEE) equation used by McCrory [13] in which:

pTEE = 7.377 − (0.073 × age) + (0.0806 × weight) + (0.0135 × height) − (1.363 × sex)(1)

Note: age (years); weight (kg); height is standing height (cm); sex (0 for males and 1 for females).

We calculated the ratio of reported energy intakes (EIrep) to pTEE to identify energy under-reporting (EIrep/pTEE < 0.4) and over-reporting (EIrep/pTEE > 1.6). We only did these analyses for children who were not breastfeeding to avoid making incorrect assumptions about the amount of breastmilk consumed.

### 2.11. Statistical Analysis

We summarized the characteristics of respondents with descriptive data analysis. We presented the energy and nutrient intakes as medians (25th and 75th percentile). We compared the baseline characteristics of children from the cross-sectional and cohort studies with Chi-square tests. The tablet- and paper-based comparisons were performed using the Mann–Whitney test for median energy and nutrient intakes, and the Chi-square test for the proportion of energy under- and over-reporting. We used the statistical software package Stata SE version 14 for all analyses. Statistical significance was set at *p* < 0.05.

## 3. Results

Among the 680 children enrolled in the cohort study, 658 (96.1%) children were successfully followed-up until they were 18 months of age. Table 2 compares the background characteristics of mothers and infants recruited into the cohort and cross-sectional sub-studies. There were no important differences in background characteristics between the two studies, which we expected as the cohort was a random sub-sample of the baseline participants.

Table 3 presents the median dietary intake of energy and nutrients for children by age group and method of data capture. The estimated median dietary intakes of energy and most other nutrients were similar across data capture methods. However, for children aged 12–18 months old, the intake of energy, niacin, folate, vitamin C and B6 was significantly higher when estimated using electronic compared to paper-based data capture. In contrast, for children aged 6–8 and 9–11 months old, the intake of calcium and vitamin A (for children aged 6–8 months old only) was significantly lower when estimated using the electronic compared to the paper-based data capture methods.

We compared the percentage of non-breastfed children with acceptable estimated intakes of energy (EIrep/pTEE) in the cohort study (*n* = 203) and cross-sectional study at the end-line (*n* = 345) (Table 4). These comparisons showed that a significantly higher percentage of children, in each age group, had acceptable energy intakes when estimated using electronic data capture compared with paper-based data capture.

## 4. Discussions

In this paper, we describe a tablet-based application developed to capture dietary data in LMICs using the multiple-pass 24-h dietary recall method. The results show it is feasible to administer a multiple-pass 24-h recall, using electronic data capture, and reliable for collecting longitudinal data for young children in a Southeast Asian country. We also demonstrate that electronic data capture can reduce energy under- and over-reporting compared with a paper-based approach. The findings suggest that the tablet-based data collection was associated with fewer subjects reported to be under-reporting, narrower energy range, and the mean nutrient intakes for calcium, iron, and zinc were likely to be more concurrent with previous findings on problem nutrients in this age group [14].

Collecting high-quality 24-h dietary recall data is resource-intensive and challenging. It relies on well-trained and skilled enumerators, but errors introduced through recall and social desirability biases often compromise the quality of the data [15]. Similar to previous studies, we found that electronic data collection improves the quality of dietary data because standardized probes built into the application guide interviewers and ensure probes are not missed [4,5,7]. However, no studies have reported findings from the use of tablet-based dietary data collection in a longitudinal study, which is one of the strengths of our study.

The integration of a food photo album, for portion size estimation, is another important feature of our tablet-based application. Although we encouraged enumerators to estimate food portion sizes by weighing actual food items whenever feasible, food pictures were helpful when actual food items were not available and for commercial (standard sized) food items.

A key innovation of the application was the immediate display of the child’s estimated intake of energy (by day and food item) at the end of the interview with a warning message if the estimated daily energy intake was either too high or too low. This feature provides an opportunity to recheck extreme energy values, thereby reducing misreporting compared with paper-based data capture. In dietary surveys, over- or under-reporting of energy intakes is a common source of error that can undermine associations between diet and disease relationships [13]. In the 39 years history of NHANES dietary data collection, a recent study found that 67.3% of women and 58.7% of men reported biologically implausible intakes of energy [16]. These levels of misreporting are comparable to those found in our study for paper-based data capture, whereas the lower levels of misreporting we found for electronic data capture were comparable or lower than those reported in a previous study using an Automated Multiple-Pass Method (AMPM) computerized interview [17]. Together these data indicate that the use of tablet-based electronic dietary data capture will enhance the quality of dietary data by reducing potential under- or over-reporting of energy intakes.

One of the challenges, when using this method, is the need for a complete FCT. Unlike food frequency questionnaires, in 24-h diet recall, respondents can recall food items that are not in the internal FCT. The current application has the flexibility to expand existing FCT by continually updating the application’s FCT during data collection. This process allows rigorous data quality checks with electronic 24-h dietary recall data capture that are not possible with paper-based data capture.

We incorporated a food photo album into our application to facilitate the estimation of food portion sizes when foods were not available to weigh. It was easy to use; however, in a large country with diverse foods, such as Indonesia, creating a comprehensive food photo album is a daunting task. Alternatively, cutting-edge technology that would not require a comprehensive food photo album, such as an automated food imaging for portion size estimation, is not yet developed for use in LMICs [18]. Thus, our approach of integrating a food photo album into an application is the best solution to facilitate portion size estimation. Indeed, the use of food photo albums is the most common technique used to estimate food portions in 24-h dietary recall platforms in Western countries [19]. We, therefore, believe it is worth investing in the development of food photo albums to facilitate dietary assessment in Indonesia and some LMICs, including a database of comprehensive atlases of food images, for standard-sized commercial foods consumed in LMICs, and customized locally specific food photo albums, for uploading into an application.

Few studies describe tablet-based dietary data collection. A recent study from Nepal recommended recording within the application of a quick food list [4]. We included the quick food list as the first pass according to the multi-pass approach, and we displayed this list on the screen, which reminded the enumerator to check whether there was any missing food before the interview ended. In this way, the application helped the respondents to recall better the food items consumed, and at the same time, helped the enumerators with the continuous display of the quick checklist throughout the interview.

We conducted this study in East Java, where the prevalence of child undernutrition is relatively high [20]. One of the main reasons for the poor nutrition status of children is poor dietary practices resulting in low intakes of key nutrients. Our findings indicated that the median intakes of key nutrients for the growth of children, such as iron, zinc, and calcium, were relatively low compared to their estimated average intake (EAR). In this study, we could not compare intakes from the same children, but the Baduta study sampling strategy ensured that children in the cross-sectional and cohort samples represented children in the study area. Our findings showed that the median energy intakes for children in the cohort sample (i.e., tablet-based) were higher than in the children from the cross-sectional sample (i.e., paper-based). The results were similar for other nutrients such as niacin, folate, vitamin B12, and vitamin C. Another study has validated a tablet-based vs. paper-based dietary intake method, but only with a group of cooperative university students [21]. No previous study has compared tablet-based vs. paper-based dietary data obtained from children under two years old, as reported here. Besides that, the intake of key nutrients such as iron, zinc, and calcium was lower than the requirements in both methods, which highlighted the poor dietary intake of children in the study area. Our findings were consistent with previous diet modeling studies, which found that calcium and iron were problem nutrients in the complementary diet in this area [14].

The major advantage of using this application is its use of a multiple-pass approach, which can improve the quality of dietary data without the use of skilled interviewers. Earlier studies using doubly labeled water to assess energy expenditure across the NHANES surveys showed that the multiple-pass approach reduced under-reporting [22]. At the same time, the application can significantly reduce the burden of data entry, which is time-consuming and often leads to data errors. Another advantage is that the application can be used for all age groups since it follows the principle of multiple-pass recall, and the user can extend the FCT according to the number of food items consumed by the population of interest. Also, we expect that users can employ the application in other countries as long as an appropriate FCT is available. There is potential to further develop tablet-based dietary data collection with automated identification of energy under- or over-reporting and the derivation of food patterns (frequency of consumption of each food item, food subgroup, and food groups) and median portion sizes for use in other software requiring this information (e.g., Optifood) [23]. The application we developed showed that it is possible to manage dietary data using a tablet for a cohort study and therefore its use should be promoted to ensure more accurate dietary data. Although a standardized dietary assessment methodology is ideal for nutrition surveillance across countries, it may take time to achieve this goal. In the meantime, we have demonstrated a reliable and efficient tablet-based 24-h dietary recall tool that can be used to collect dietary data for large-scale surveys and nutrition research.

## Figures and Tables

**Table 1:** Acceptable energy ranges used to identify potential energy under- or over-reporting.

	Breastfed Infants (kcal)	Non–Breastfed Infants (kcal)
6–8 months old	100 to 600	400 to 800
9–11 months old	200 to 750	500 to 900
12–23 months old	500 to 1100	600 to 1200

**Table 2:** Background characteristics of mothers and children under two years.

Characteristics of Mother	Cohort		Cross-Sectional		*p*-Value ^1^
	Number	(%)	Number	(%)	
**Age of Mother (year)**					
≤15	1	0.1	2	0.1	0.006
15–19	58	8.5	111	4.0	
20–29	337	49.7	1298	47.4	
30–39	261	38.5	1154	42.1	
>40	21	3.1	175	6.4	
**Highest Level of Education**					
Never attended any school	15	2.2	38	1.4	0.102
Completed primary school	161	23.7	479	17.7	
Completed junior high school	215	31.7	661	24.4	
Completed senior high school	238	35.1	1111	41.1	
Academy/D1/D2/D3	49	7.2	415	15.3	
**Main Occupation**					
Housewife	532	78.5	2,049	74.8	0.024
Government employee	42	6.2	282	10.3	
Entrepreneur/trader	46	6.8	179	6.5	
Factory labor	34	5.0	69	2.5	
Other	24	3.5	161	5.9	
**Household Food Security**					
Food secure	505	74.5	2179	79.5	0.05
Food insecure without hunger	158	23.3	441	16.1	
Food insecure with hunger	15	2.2	120	4.4	
**Wealth Index**					
Least	140	20.7	582	21.5	0.96
Second	173	25.5	637	23.6	
Middle	104	15.3	420	15.5	
Fourth	141	20.8	605	22.4	
Highest	120	17.7	459	17.0	

^1^ Chi-square to test for independence to assess any significant difference between treatment groups. *p* < 0.05 indicates statistical significance.

**Table 3:** Dietary intake of cross-sectional (paper-based) and cohort (tablet-based) sub-studies among breastfed children ^1^.

Intake	Age Group	Paper-Based Data Collection		Tablet-Based Data Collection		*p*-Value ^2^
		Median	(IQR)	Median	(IQR)	
Energy (Kcal)	6–8 months	174.3	103.5–290.1	177.9	112–295.2	0.416
	9–11 months	297.7	185.4–444.4	336.0	207–490.7	0.135
	12–18 months	481.4	315.6–702.4	555.4	371.1–783.3	0.004
Protein (g)	6–8 months	4.1	2.4–7.0	4.9	2.6–7.9	0.09
	9–11 months	9.0	4.7–13.1	10.2	5.8–15.6	0.146
	12–18 months	15.2	9.7–23.9	17.0	10.4–24.9	0.185
Calcium (mg)	6–8 months	113.2	39.5–194.7	71.8	29.3–152.4	0.009
	9–11 months	92.7	42.8–217.5	76.6	39.3–132	0.046
	12–18 months	141.9	75.8–262.5	124.9	75–241.4	0.409
Iron (mg)	6–8 months	2.1	1.0–3.7	1.6	1.0–3.0	0.23
	9–11 months	2.3	1.1–4.4	2.3	1.4–3.8	0.583
	12–18 months	3.3	1.8–5.2	3.4	2.0–5.5	0.652
Zinc (mg)	6–8 months	0.9	0.4–1.7	0.9	0.5–1.8	0.524
	9–11 months	1.4	0.7–2.5	1.7	0.9–2.9	0.073
	12–18 months	2.3	1.5–3.5	2.5	1.4–3.9	0.392
Vitamin A (retinol)	6–8 months	78.8	4.4–142.9	42.4	2.5–115.5	0.037
	9–11 months	39.6	3.4–139.8	25.3	3.9–114.8	0.328
	12–18 months	96.3	6.9–236.1	77.2	11.2–177.9	0.278
Thiamine (mg)	6–8 months	0.1	0.1–0.2	0.1	0.1–0.2	0.736
	9–11 months	0.1	0.1–0.3	0.2	0.1–0.3	0.293
	12–18 months	0.3	0.2–0.5	0.3	0.2–0.5	0.051
Riboflavin (mg)	6–8 months	0.1	0.1–0.3	0.1	0.1–0.2	0.881
	9–11 months	0.2	0.1–0.3	0.2	0.1–0.4	0.229
	12–18 months	0.3	0.2–0.6	0.3	0.2–0.6	0.468
Niacin (mg)	6–8 months	1.4	0.9–2.5	1.4	0.8–2.2	0.601
	9–11 months	2.1	1.1–3.3	2.3	1.3–3.5	0.169
	12–18 months	3.0	1.8–4.8	3.5	2.1–5.8	0.037
Vitamin B6 (mg)	6–8 months	0.2	0.1–0.4	0.2	0.1–0.4	0.744
	9–11 months	0.3	0.2–0.4	0.3	0.2–0.5	0.024
	12–18 months	0.4	0.3–0.6	0.5	0.3–0.7	0.005
Vitamin B12 (mg)	6–8 months	0	0–0.2	0.1	0–0.5	<0.001
	9–11 months	0.2	0–0.7	0.5	0.1–1.0	0.002
	12–18 months	0.6	0.2–1.2	0.6	0.3–1.3	0.271
Folate (DFE)	6–8 months	11.1	3.2–28.9	18.4	6.1–37.8	0.001
	9–11 months	27.7	14.4–51.4	30.5	16.6–57.9	0.158
	12–18 months	47	27.8–76.2	52.6	31.1–82.6	0.033
Vitamin C (mg)	6–8 months	12.0	5.0–22.0	9.0	3.4–19.6	0.15
	9–11 months	8.3	2.3–24.1	7.5	1.8–16.4	0.299
	12–18 months	8.6	2.8–22.8	11.3	4.1–30.2	0.012

^1^ Nutrient intakes presented as median (25–75th percentile interquartile range). ^2^ Somers’ D test-adjusted for cluster sampling. *p* < 0.05 indicates statistical significance.

**Table 4:** Acceptable energy reported by paper-based and tablet-based dietary data ^1^.

Age Group	Acceptable Energy Intake Reported by Paper-Based Data Collection		Acceptable Energy Intake Reported by Tablet-Based Data Collection		*p*-Value ^2^
	%	N	%	N	
6–8 month	40.9	36	60.8	31	0.018
9–11 month	56.9	62	72.4	42	0.035
12–18 month	54.3	75	80.7	71	<0.001

^1^ Acceptable energy intake calculated by McCrory’s method [13]. ^2^ Chi-square to test for independence to assess any significant difference between treatment groups. *p* < 0.05 indicates statistical significance.
